# Evolutionary plasticity and functional versatility of CRISPR systems

**DOI:** 10.1371/journal.pbio.3001481

**Published:** 2022-01-05

**Authors:** Eugene V. Koonin, Kira S. Makarova

**Affiliations:** National Center for Biotechnology Information, National Library of Medicine, National Institutes of Health, Bethesda, Maryland, United States of America

## Abstract

The principal biological function of bacterial and archaeal CRISPR systems is RNA-guided adaptive immunity against viruses and other mobile genetic elements (MGEs). These systems show remarkable evolutionary plasticity and functional versatility at multiple levels, including both the defense mechanisms that lead to direct, specific elimination of the target DNA or RNA and those that cause programmed cell death (PCD) or induction of dormancy. This flexibility is also evident in the recruitment of CRISPR systems for nondefense functions. Defective CRISPR systems or individual CRISPR components have been recruited by transposons for RNA-guided transposition, by plasmids for interplasmid competition, and by viruses for antidefense and interviral conflicts. Additionally, multiple highly derived CRISPR variants of yet unknown functions have been discovered. A major route of innovation in CRISPR evolution is the repurposing of diverged repeat variants encoded outside CRISPR arrays for various structural and regulatory functions. The evolutionary plasticity and functional versatility of CRISPR systems are striking manifestations of the ubiquitous interplay between defense and “normal” cellular functions.

## Abstract 2

The CRISPR systems show remarkable functional versatility beyond their principal function as an adaptive immune mechanism. This Essay discusses how derived CRISPR systems have been recruited by transposons on multiple occasions and mediate RNA-guided transposition; derived CRISPR RNAs are frequently recruited for regulatory functions.

## Introduction

The CRISPR systems are known primarily as a new generation of genome editing tools that possess unprecedented specificity and efficiency thanks to the use of guide RNAs to recognize unique sequences in the genome [1–3]. This specificity of nucleic acid recognition also underlies the primary biological function of CRISPR in prokaryotes, namely adaptive immunity against viruses and other mobile genetic elements (MGEs) [4–7]. The CRISPR immune response involves 3 stages:

- adaptation, the process of acquisition of pieces of foreign DNA (protospacers) that become spacers inserted between repeats in CRISPR arrays and are subsequently employed to produce guide RNAs that specifically target the cognate foreign nucleic acid;
- expression and maturation of the CRISPR (cr) RNAs, whereby the long transcript of the CRISPR array, the pre-crRNA, is processed to yield functional, mature crRNA; and
- interference, whereby crRNAs are exploited as guides to recognize the target DNA or RNA that is then, typically, cleaved by the nuclease moiety of the CRISPR effector complex.

Each of these stages in the CRISPR immune function is mediated by a distinct set of Cas (CRISPR associated) proteins that comprise functional modules of the CRISPR systems [8,9]. Characteristically of defense systems [10], CRISPRs are prone to fast evolution that involves not only sequence change but also numerous rearrangements and replacements of the Cas protein complexes [11,12]. In addition to the 3 core modules, most of the CRISPR systems also encompass various accessory genes, which encode proteins modulating the activity of the core CRISPR components in manners that, with a few exceptions, remain poorly characterized [13,14].

From the early days of CRISPR research, it seemed natural to surmise that CRISPR systems would be recruited for various nondefense functions, including regulation of gene expression, but probably much more [15,16]. However, more than a decade later, the information on nondefense functions of CRISPR remains scarce. Nevertheless, one remarkable theme has emerged prominently, namely the recruitment of partially degraded, defective CRISPR systems by transposons that employ them for RNA-guided transposition [17,18]. In addition, a variety of highly derived *cas* operons have been discovered that are often not linked to CRISPR arrays [19,20]; their functions remain enigmatic and await experimental study. Another trend that is becoming increasingly prominent is the repurposing of the CRISPR themselves, that is, crRNAs and their derivatives, for various, primarily regulatory functions rather than for target recognition followed by cleavage [21–23].

In this essay, we outline our current understanding of the evolutionary plasticity and functional versatility of CRISPR. Evolutionary plasticity refers to gain and loss of components by CRISPR systems as well as functionally consequential evolutionary changes in *cas* genes, for example, those that lead to enzyme inactivation. These evolutionary changes give rise to remarkable functional versatility, that is, a broad repertoire of biological functions and molecular mechanisms across the numerous variants of CRISPR systems and their derivatives. The universe of CRISPR is expanding fast with advancing genome and especially metagenome sequencing, but we believe that general trends can already be captured.

### Functional versatility of CRISPR shaped by exaptation and diversification

In their principal role as adaptive immunity mechanisms against viruses and other foreign DNA (and in some case, RNA), CRISPR systems show remarkable functional versatility [11]. This multitude of functionalities was apparently shaped by exaptation [24,25], that is, recruitment of various genes with nondefense functions as CRISPR system components, as well as extensive diversification [16,26].

**Fig 1:**
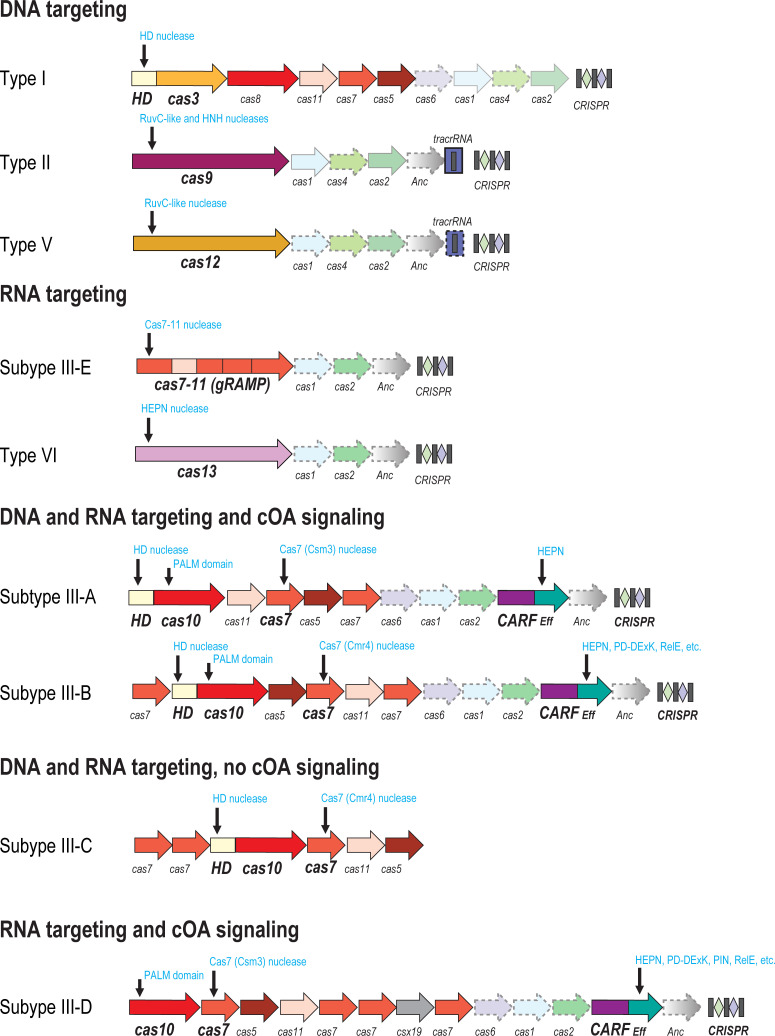
Organizational and functional diversity of CRISPR as adaptive immune systems. General organization of loci encoding different CRISPR-Cas systems is shown. The protein-coding genes are denoted by arrows (not to scale). Homologous genes are shown by the same color. The arrows with dashed outline identify genes that are optional in the respective loci. Adaptation module genes are semitransparent. Gene names are indicated according to the established nomenclature [11]. Vertical arrows indicate genes that are directly involved in target cleavage or cOA synthesis. Anc, ancillary gene; CARF, CRISPR-associated Rossmann fold; Eff, effector domain; PALM, PALM domain involved in cOA synthesis; RuvC-like, HNH, HEPN, HD, PD-DExK, PIN, RelE, nucleases of the respective families.

Classification of CRISPR systems is based primarily on the composition of the expression and interference modules (hereafter, jointly denoted effectors) of *cas* genes in the respective CRISPR-*cas* loci. They are divided into 2 classes, 6 types, and over 30 subtypes (and counting) [11]. The adaptation module is relatively uniform across CRISPR types and subtypes (although see below on a major variation in type III), but the effector modules vary extensively (Fig 1). The most drastic divide is between Class 1 and Class 2: In Class1, the processing and interference modules are multisubunit complexes formed by several Cas proteins (known as Cascade in the case of type I systems), whereas in Class 2 all these activities are combined within a single multidomain protein. The Class 1 and Class 2 effectors are unrelated and have completely different evolutionary histories [27].

The effector complexes of type I and type III appear to be homologous, but the similarity between them is distant and became fully apparent only after the structures of both types of complexes were resolved [9,28]. In both these CRISPR types, the scaffold of the effector complex is formed by several paralogous proteins (Cas5 and Cas7) containing a derived RNA recognition motif (RRM) domain. The complexes of both types also contain the so-called large subunits (Cas8 and Cas10 in types I and III, respectively) that may or may not be homologous and small subunits (Cas11), which are homologous despite vanishing sequence conservation, as demonstrated by structure comparison [29]. Cas10 is an RNA polymerase of the Palm domain superfamily (another distinct variety of the RRM fold) that specifically synthesizes cyclic oligoadenylates (cOAs) [30,31], although in subtype III-C, the polymerase is inactivated by substitution of some of the catalytic amino acid residues [9]. By contrast, Cas8, also a large protein, contains no recognizable catalytic domains and is not significantly similar to Cas10.

The RNA polymerase activity of Cas10 was predicted in early analyses of Cas proteins [32], but the experimental validation lagged behind for more than a decade, and the function of the highly conserved catalytic domain remained an enigma. The solution opened a new chapter in the study of CRISPR through the discovery of a signal transduction pathway embedded within type III systems [30,31,33]. When a bacterium or an archaeon carrying a type III locus is infected by a familiar virus, for which there is cognate spacer, target recognition activates the polymerase activity of Cas10, which is both a structural subunit of the effector complex and an active enzyme. As a result, cOA is produced and binds to the CRISPR-associated Rossmann Fold (CARF) domain of an “accessory,” but nearly ubiquitous among type III systems, protein, such as Csm6 or Csx1. cOA binding allosterically activates the second domain of this protein, an RNase of the HEPN superfamily. A considerable sequence and structural diversity of sensor CARF domains exists across type III systems, suggestive of variations in the signaling pathways and the possibility that they use different signal oligonucleotides, which remain to be characterized [34]. Furthermore, the nuclease moiety of the cOA-activated CRISPR effector also varies. Although HEPN is most common, other nucleases, such as those of the restriction endonuclease fold, are also induced by cOA to indiscriminately cleave single-stranded RNA (ssRNA) and single-stranded DNA (ssDNA) [35–37]. Thus, the cOA-activated defense pathway displays substantial combinatorial complexity, the functional implications of which remain to be explored [33].

The cOA pathway couples 2 fundamentally distinct forms of antivirus response that appear to be coupled in various defense systems: direct attack on an invading foreign element and induction of cell dormancy or programmed cell death (PCD) [38]. Once a virus infects a cell containing a type III system with a cognate spacer, the CRISPR effector recognizes the protospacer and cleaves both the virus genome itself, via the HD nuclease domain of Cas10, and transcripts of the virus genome, via a Cas7 subunit of the effector. Simultaneously, the HEPN nuclease activated by cOA cleaves RNA molecules nonspecifically, resulting in growth arrest or PCD, which represent altruistic form of defense whereby a cell arrests its own growth or even commits suicide to prevent infection of other cells in the population [39,40]. The altruistic mechanism is thought to be activated when the cells senses the failure of immunity [38] or alternatively might function preventively as a backup. The complexity of signal transduction built into type III systems does not stop here. The indiscriminate RNase activity of HEPN nucleases is a costly reaction, so type III systems are endowed with a dedicated mechanism to mitigate the damage whereby cOA is cleaved by RING nucleases encoded either within the CRISPR loci or in different genomic locations [41]. The RING nucleases show considerable structural diversity; some are distinct versions of the CARF domain, whereas others are enzymes of different families [34,41]. The details of the interplay between immunity and dormancy/PCD in the CRISPR response, which are likely to differ depending on the lifestyles of bacteria and archaea, remain to be characterized experimentally.

The cOA signaling pathway is an integral, key component of type III CRISPR systems, although some of these have secondarily lost it through the elimination of the CARF-HEPN protein and inactivation of Cas10, which in these cases retains its structural role only. There is much additional complexity and versatility associated with type III systems, as demonstrated by systematic analysis of genomic neighborhoods for genes that are significantly associated with CRISPR [13,14]. Almost none of these genes have been experimentally characterized. Computational predictions reveal recurrent connections of type III systems with various signal transduction pathways as well as membrane transport. A lot of novel biology will undoubtedly be discovered when the interactions of these accessory proteins with the core CRISPR machinery are studied experimentally.

Apart from the functional versatility of the effectors, there is less pronounced but functionally relevant variation in the adaptation modules of type III CRISPR systems as well. In particular, numerous type III variants include a reverse transcriptase, likely derived from group II introns, which is typically fused to Cas1 protein and mediates spacer incorporation by reverse transcription of virus RNAs [42–44].

**Fig 2:**
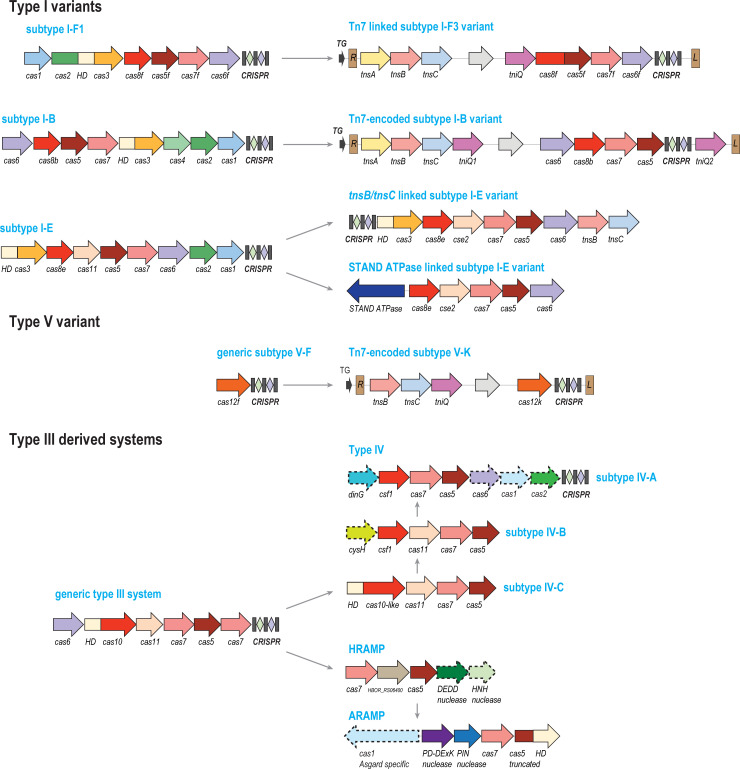
Exaptation of derived CRISPR systems for functions distinct from adaptive immunity. Designations of protein-coding genes and *cas* gene names are the same as in Fig 1. The thin arrows pointing from the schematics in the left part of the figure to those in the right part indicate the inferred directionality of evolution, from stand-alone CRISPR systems to derived ones associated with transposons or STAND NTPases. Left (L) and right (R) ends of the IS elements are shown as brown rectangles, respectively and are not to scale. The terminal integration site (TG) is shown by a small dark gray arrow. The gray arrow generically denotes transposon cargo genes. The Tn7 transposon genes (*tnsA*, *B*, *C*, and *tniQ*) are designated according to the established classification [69]. DEDD, nuclease of the respective family; other abbreviations are the same as in Fig 1.

A far more drastic alteration of the adaptation module might have occurred in subtype I-E systems of various bacteria in the family Streptomycetaceae [17]. These loci contain a CRISPR array and appear to be competent for interference but lack the typical adaptation module (that is, *cas1* and *cas2* genes). Instead, they are consistently associated with *tnsB* and *tnsC*, the genes encoding 2 subunits of the Tn-like transposon transposase (Fig 2). Given that transposon ends were not detected in the vicinity of these loci, the hypothesis has been proposed that the transposase has been recruited as an alternative adaptation module [17]. Given the apparent origin of the Cas1 protein, the integrase in the classical CRISPR adaptation module, by exaptation of the transposase of a distinct transposon family, the casposons [45,46], parallel evolution of an alternative adaptation module from a distinct transposase appears plausible.

**Fig 3:**
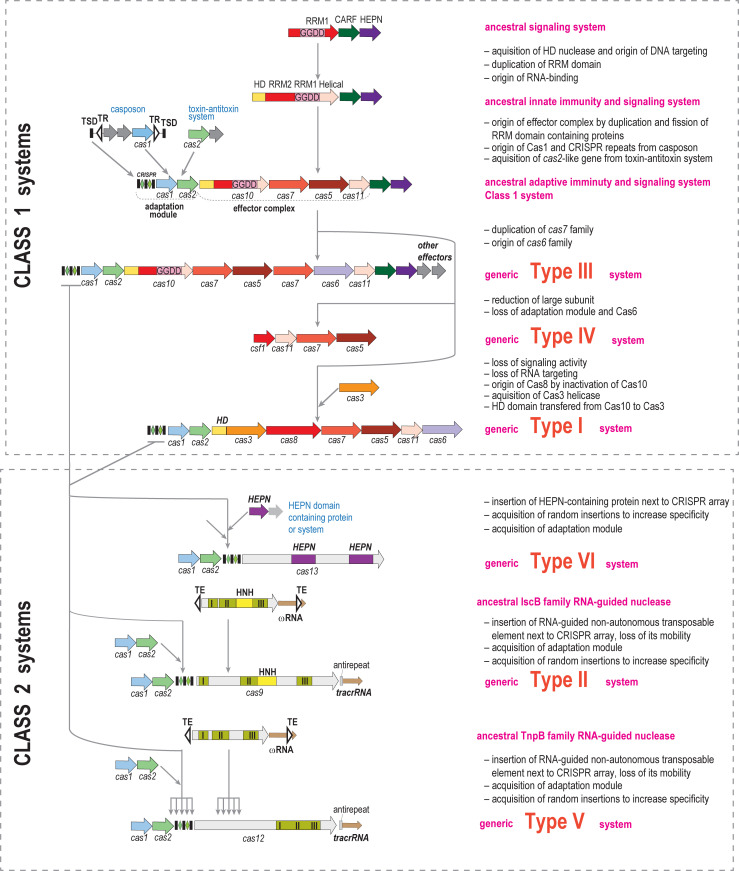
Origins and evolution of CRISPR-Cas systems: Initial accretion of components and subsequent reduction. The figure schematically shows the hypothetical evolutionary scenarios for the common varieties of CRISPR systems and their derivatives. Genes are shown by block arrows not drawn to scale. Protein and domain families are denoted by color. The evolutionary events thought to have been involved in each step are briefly described to the right of the schematics. Inverted repeats flanking transposable elements (IscB and InpB) are shown by triangles. The multipronged arrows pointing to type V indicate the origin of the effector genes of different subtypes from different families of TnpB as well as independent origins of the adaptation modules. The 3 distinct sequence motifs that comprise the catalytic site of the RuvC-like nucleases in IscB, Cas9, TnpB, and Cas12 are denoted I, II, and III.

Notably, all these organizational and functional complexities, and especially, the integration of CRISPR adaptive immunity with a fundamentally different form of defense, dormancy induction/PCD, through a dedicated signal transduction pathway, are characteristics of type III but not type I systems. This appears surprising given that type I CRISPRs are considerably more abundant among bacteria and archaea, and hence, apparently, more evolutionarily successful than type III [11]. Although there is currently no direct evidence that the coupling of adaptive immunity with dormancy induction/PCD is advantageous to bacteria and archaea carrying type III CRISPR, the conservation of the cOA pathway in most type III variants implies such an advantage. Tracing evolutionary connections of the signal transduction machinery provides a clue for the ultimate origin of the Class 1 effectors (Fig 3). Search of bacterial genomes for homologs of Cas10 identified a putative operon that consists of a gene encoding a “minimal” Cas10 homolog consisting of the polymerase domain alone and a protein comprising a fusion of CARF and HEPN domains [47]. So far, this module has not been studied experimentally, but the domain composition implies that it functions as an abortive infection (ABI) system that, after cOA synthesis is triggered by virus infection, causes dormancy/PCD through the nonspecific RNase activity of HEPN. Given the simplicity and compactness of this type of ABI module, they appear to be likely ancestors of the type III effectors (as opposed to the reverse direction of evolution). Subsequent evolution of CRISPR would involve major complexification of type III effector modules including capture of additional domains, such as the HD nuclease domain of Cas10, as well as likely serial duplication of the RRM domain of Cas10 yielding the entire superfamily of RRM-containing Cas proteins: Cas5, Cas6, and Cas7 (collectively denoted repeat-associated mysterious proteins, RAMPs) [27]. Under this scenario, type III CRISPR systems were the first to evolve, whereas Type I systems are derived forms that have lost the cOA signaling circuit and hence the coupling of target recognition and cleavage with dormancy/PCD. Thus, a major trend in the evolution of Class 1 CRISPR systems, after the initial phase of accretion of *cas* genes, apparently, was functional reduction and simplification (Fig 3). Below we discuss even more dramatic manifestations of this trend.

The evolutionary scenarios for the Class 2 CRISPR effectors are completely different [48] and present striking cases of exaptation of proteins encoded by MGE for defense functions in CRISPR systems. The single-protein effectors of type II and several subtypes of type V and type VI all appear to have evolved independently. The type II and type V effectors (Cas9 and Cas12, respectively) are both homologous to transposon-encoded proteins known, respectively, as IscB and TnpB, which are abundant in many bacteria and archaea. The role of IscB and TnpB in the transposon life cycle remains unknown. Recently, however, it has been shown that these proteins are RNA-guided nucleases that utilize a small RNA encoded by an adjacent gene (denoted ωRNA, after Obligate Mobile Element Guided Activity, OMEGA) to target specific sequences in a manner resembling the CRISPR mechanism [49,50]. All these proteins contain homologous RuvC-like nuclease domains, but, likely, specific evolutionary relationships can be traced. The Cas12 proteins of different type V subtypes appear to have evolved independently from different families of TnpB as inferred from strongly supported affinities in the phylogenetic trees of the TnpB family [48]. Cas9 proteins, by far the most common Class 2 effectors, share a distinct domain architecture with IscB, namely an HNH nuclease domain inserted into the RuvC-like nuclease, suggesting that IscB could be the ancestor of Cas9 [51]. In a phylogenetic tree rooted by the recently discovered IsrB proteins, homologs of IscB and Cas9 that lack the HNH domain, the Cas9 branch, is lodged with IscB, supporting this ancestral relationship [49].

The type VI effectors, Cas13 proteins, are unrelated to the type II and type V effectors and contain 2 HEPN domains, which are both catalytically active RNases required for the Cas13 function. Type VI systems are the only known variety of CRISPR that is strictly specific for RNA targets [52]. Furthermore, unlike type I, II, and V systems that target only DNA, but similar to type III systems that cleave also RNA, type VI CRISPR, once activated by target recognition, cleave RNA indiscriminately and induce dormancy or cell death [53]. The major difference from type III is that type VI systems are much simpler: Both the RNA-guided cleavage of the target and the nonspecific RNA cleavage are performed by the same catalytic domain, and there is no built-in signal transduction circuit controlling cell damage. In essence, type VI CRISPR systems are target-specific, RNA-guided ABI modules. The HEPN domains of Cas13 proteins are homologous to the HEPN domains of the toxin RNases of numerous toxin–antitoxin (TA) systems including ABI, but the presence of 2 HEPN domains within the same protein appears to be a unique feature of Cas13. Although HEPN domains are too divergent for robust phylogenetic analysis, the widespread and simple architecture of the toxin RNases contrast the rarity and complexity of Cas13, suggesting that type VI CRISPR evolved from an ABI system [11,17,27].

All Class 2 CRISPR effectors are much larger proteins than their likely ancestors, so their evolution appears to have involved accretion of additional domains. This seems to have been a gradual, piecemeal process because apparent evolutionary intermediates between TnpB and typical type V effectors (Cas12) that are larger than the former but smaller than the latter have been identified [48,54]. Such enlargement and complexification seem to be a general trend, having occurred convergently in the evolution of multiple CRISPR effectors, including Cas9, Cas10, Cas12, and Cas13 (Fig 3).

The opposite trend, secondary partial degradation, and simplification of effector modules are recurrent as well. We note above that type I CRISPR systems might have evolved from type III along this route. An even more striking case of reductive evolution is presented by the recently discovered subtype III-E [11,55,56]. The III-E effector presents an appearance of a paradox: Although clearly derived from type III ancestors, it formally could be classified as Class 2 inasmuch as it is a single protein that comprises a fusion of 4 Cas7 subunits and Cas11 (hence the designation Cas7–11, also known as g(iant)RAMP). Subtype III-E systems lack some of the functionality of the “regular” type III systems: Having lost the *cas10* gene, they neither cleave the target DNA nor induce dormancy/PCD via the cOA pathway. Their only remaining route of interference is target RNA cleavage by the Cas7 domains. Furthermore, unlike the rest of type III systems, in which the crRNA maturation is catalyzed by the dedicated Cas6 protein, but similarly to the type VI effector Cas13, Cas7–11 itself catalyzes this reaction, in addition to target RNA cleavage [56]. The evolutionary intermediate between typical type III systems and the derived subtype III-E appears to be variant III-D2 that retains Cas10 and Cas6 but also encompasses a protein with 3 fused Cas7 domains (Cas7x3) [56].

The brief overview given in this section inevitably falls short of capturing all the complexity and functional versatility of the defense molecular machinery of CRISPR systems. Hopefully, however, it is sufficient to highlight the general trends of the evolution of the CRISPR diversity that are becoming apparent, namely the convergent routes of initial complexification via accretion of proteins and domains, in many cases recruited from MGE, and subsequent reductive evolution that yields a plethora of specialized variants (Fig 3). The exaptation of MGE components for roles in CRISPR systems is a clear manifestation of the “guns for hire” phenomenon, whereby the same molecular components (in many cases, nucleases) are employed both by hosts as means of defense and by MGE, as offense or antidefense weapons [57]. In the next section, we address even more drastic modifications of CRISPR systems that involve their exaptation for functions distinct from adaptive immunity.

### The exaptive splendor of CRISPR

In this section, we discuss the well characterized as well as tentative cases of exaptation of CRISPR systems and their components, that is, their recruitment for alternative biological functions distinct from bacterial or archaeal adaptive immunity [16,26]. Diverse cases of CRISPR exaptation have been discovered (and counting), although they seem to comprise only a relatively small minority of CRISPR systems [11]. In some instances, an intact, apparently functional CRISPR systems is subverted for a new function. The *dev* operon of Myxobacteria that regulates the sporulation process encodes a typical subtype I-B CRISPR system including the adaptation module and the CRISPR array [58,59]. However, this CRISPR system contributes to the regulation of sporulation via a mechanism that is distinct from the typical CRISPR activity. It has been shown that the complex of Cas5, Cas7, and Cas8 proteins, a subcomplex of the Cascade, employs a distinct antisense RNA to abrogate the expression of *devI*, a sporulation inhibitor, and thus promote sporulation [59]. Most likely, the I-B CRIPSR system in the *dev* operon performs a dual function, in both adaptive immunity and expression regulation. Without targeted experimental studies, it is impossible to tell how common the dual functionality of CRISPR systems might be, opening the tantalizing possibility that such secondary recruitment occurred in many cases. Apart from the utility for the host cell, the gain of a new function that is important for the host could make it addicted to CRISPR, preventing the loss of the CRISPR locus and stabilizing the “symbiosis” between CRISPR and the host organism (more on such addiction below).

Other cases of repurposing of intact, functional CRISPR systems have been identified in viruses. Some bacteriophages infecting *Vibrio* bacteria encode a subtype I-F CRISPR system that targets innate immunity systems of the host bacteria and thus contributes to the virus–bacterium arms race [60,61]. CRISPR systems have also been discovered in multiple megaphages that infect Bacteroidetes [62]. This is a striking illustration of the guns for hire principle. Below we discuss several additional cases of this form of exaptation, which is a persistent trend in the evolution of CRISPR derivatives (Fig 3).

On many other occasions, exaptation of CRISPR involves derived forms resulting from reductive evolution. The first case in point are type IV systems, another reduced derivative of type III (Fig 2). Type IV systems possess homologs of Cas7 and Cas5 but lack Cas10 (or Cas8) and instead encompass a much smaller protein, which plays the role of the large subunit (Csf1) of the effector complex but does not contain any recognizable enzymatic domains [63–65]. Notably, in the recently discovered subtype IV-C, Csf1 protein contains a carboxyl-terminal HD nuclease domain related to the corresponding domain of Cas10. In the phylogenetic tree of Cas7, subtype IV-C is the deepest branch, suggesting that it could represent an intermediate stage of evolution of type IV from some variant of type III. Comparisons of the solved structures of the effector complexes also support the affinity of type IV and III [64,66]. The subtypes within type IV show considerable variability of gene composition. In particular, subtypes IV-A and IV-C encompass Cas11, the small subunit of the effector complex, whereas subtype IV-B systems lack this protein. Additionally, different subtypes have distinct accessory proteins, such as the DinG helicase in subtype IV-A and the inactivated homolog of APS/PAPS reductase CysH in subtype IV-B and some subtype IV-A systems. The presence of these genes that are tightly associated with type IV loci suggests distinct functionalities that remain uncharacterized, although it has been shown that DinG is required for subtype IV system interference activity against plasmids [67]. All identified type IV systems are located on plasmids, integrative conjugative elements (ICEs), prophages, and some free phages. Those spacers in CRISPR arrays of subtypes IV-A and IV-C, for which protospacers were detected, mapped primarily to genes of plasmid conjugative machinery [65]. Thus, type IV systems are most likely engaged in competition among MGE, in particular plasmid exclusion. The molecular mechanisms of their action, however, remain enigmatic given that, with the exception of the rare subtype IV-C, they all lack recognizable nuclease domains. Furthermore, there are major functional differences between subtypes IV-A and IV-C, on the one hand, and IV-B, on the other hand. The IV-A effector complexes that lack Cas11 bind crRNA similarly to other CRISPR systems, whereas subtype IV-B systems bind heterogeneous small RNAs via a filament formed by Cas11 subunits [29], suggestive of a distinct, unknown mechanism. Regardless of the mechanistic details, type IV systems are another clear-cut case of CRISPR recruitment as “guns for hire.”

The next variety of derived CRISPR systems we discuss is an even more striking exhibit for the same principle. A genomic survey of CRISPR systems has shown that numerous Tn7-like transposons encode partially degraded subtypes I-B, I-F, and V-K CRISPR loci (Fig 2) [17,68,69]. These CRISPR systems have been acquired by transposons on at least 3, but probably, more independent occasions, suggesting that they confer selective advantages on the transposons. Furthermore, all transposon-encoded CRISPR systems have lost the interference capacity albeit via different routes, either by losing the *cas3* genes, which encodes the helicase–nuclease protein that shreds DNA in type I systems, or by inactivation of the catalytic site of the RuvC-like nuclease in V-K. Hence, it has been proposed that this variety of CRISPR mediates RNA-guided transposition, a phenomenon that has not been described prior to these observations [68]. Indeed, this route of transposition has been experimentally demonstrated for all major varieties of transposon-encoded CRISPR systems, subtypes V-K, I-B, and I-F [70–73]. In each case, the effector complex of the CRISPR systems binds to the target via the guide crRNA and delivers the transposase which inserts the transposon within a short distance from the recognized protospacer. Accordingly, the RNA-guided transposition systems were dubbed CRISPR-associated transposase (CAST). Notably, in the case of CAST-V-K and CAST-I-F, all transposition appears to be RNA-guided (some details of the molecular mechanism are discussed below), whereas transposons encoding CAST-I-B alternate 2 modes of transposition, one of which is CRISPR independent [70].

Type IV CRISPR systems, as well as the CASTs, deviate from the adaptive immune function of CRISPR, but are typically associated with CRISPR arrays and rely on the same fundamental molecular principle, namely employing guide RNAs to ensure the specificity of target recognition. Other CRISPR derivatives seem to depart from the mainstream even further (Fig 2).

In numerous species of *Streptomyces*, a derived subtype I-E system consisting of *cas5*, *cas6*, *cas7*, and *cas8* genes colocalizes with a gene coding for a STAND NTPase, a putative PCD effector [14]. Given the absence of Cas3, an adaptation module or an array, this module cannot be a typical CRISPR system, but rather, can be predicted to function as a distinct defense mechanism, in conjunction with the STAND NTPase.

Many Haloarchaea encode a distinct, highly diverged CRISPR derivatives dubbed haloarchaeal RAMP (HRAMP) that encompass homologs of Cas5 and Cas7 along with additional nucleases and uncharacterized proteins (Fig 2) [20]. In the case of HRAMP, the sequences of the Cas5 and Cas7 homologs have diverged to such an extent that tracing their origin to a specific subtype or even type of regular CRISPR systems is challenging, although there is somewhat higher similarity to the homologs from type III systems. The presence of apparently active nucleases implies that HRAMP is a distinct defense system that, however, can be expected to function via mechanisms distinct from those of CRISPR. CRISPR system derivatives distantly related to HRAMP have been identified in genomes of many Asgard archaea (and accordingly denoted ARAMP) [19]. In addition to the components distantly related to those of HRAMP, most of the ARAMPs encompass a distinct form of the adaptation integrase Cas1, making their mechanism even more enigmatic (Fig 2). Furthermore, Asgard genomes encode an unprecedented diversity of Cas1 homologs, some possibly associated with novel MGE [19].

All the CRISPR-like systems discussed here are limited in their spread to a particular group of prokaryotes or MGE, which supports the view that these are derived CRISPR forms. There is little doubt that, with further advance of genomics and metagenomics, many additional specialized CRISPR derivatives will be discovered.

### CRISPR RNAs exapted for new functions

**Fig 4:**
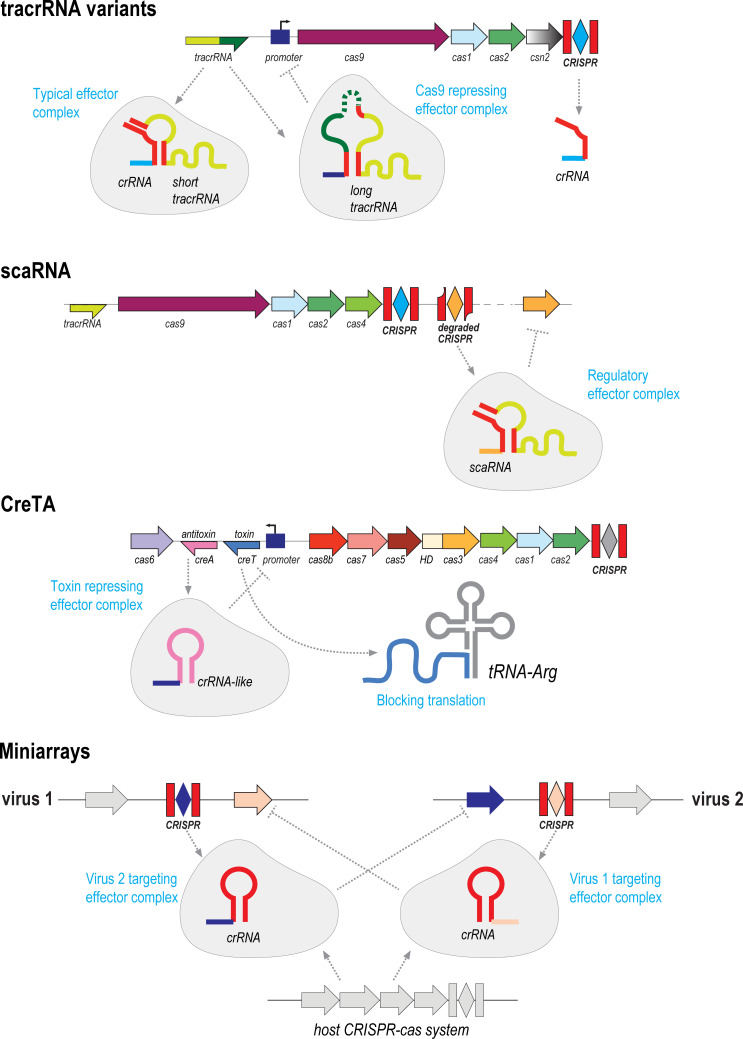
Exaptation of CRISPR repeats for regulatory functions. Schematic representation of the functions of repurposed CRISPR repeats. Designations are the same as in Fig 1.

In the final section of this essay, we discuss a different type of exaptation of CRISPR system components, namely repurposing of the crRNAs or just the repeats, often for additional functions within CRISPR systems (Fig 4). The best known case are the *trans*-acting crRNAs (tracrRNAs) that are required for interference in type II and some subtypes of type V systems [74–77]. The tracrRNAs consist of an antirepeat and a distal portion that contains a Rho-independent transcription terminator. The function of the tracrRNA is to facilitate maturation of crRNA catalyzed by RNase III and to form the functional guide RNA through duplex formation between the antirepeat and one of the repeats of the crRNA. In the guide RNA complex, the structural features of tracrRNA ensure maximum exposure of the spacer [75]. The tracrRNAs are encoded within CRISPR loci but not contiguously with the array. Detailed phylogenetic analysis of tracrRNAs from 2 groups of bacteria suggests that they evolved independently in different CRISPR variants, via duplication and relocation of a repeat [75].

A distinct, long form of tracrRNA (L-tracrRNA) present in many *Streptococcus* species has remarkably been recently shown to regulate the expression of the II-A *cas* operon [21]. Similarly, to the short-form tracrRNA (S-tracrRNA), L-tracrRNA contains an antirepeat and forms the guide RNA complex with a crRNA. Additionally, L-tracrRNA contains a 9-bp sequence that is complementary to the promoter of the cas operon and by base pairing with the promoter directs Cas9 to repress transcription about 3,000-fold, as compared to a mutant with deleted L-tracrRNA coding sequence. Such regulation lowers the efficiency of adaptive immunity but also substantially reduces the cost of CRISPR due to autoimmunity [21]. Thus, in this case, a duplicated (anti)repeat, is repurposed for autoregulation as part of the L-tracrRNA.

Similar repurposing of an ectopic copy of a repeat and of Cas9 has been studied in detail in the pathogenic bacterium *Francisella novicida*. Here, Cas9, in a complex with tracrRNA and sca(Small CRISPR-Associated)RNA, which is another small RNA encoded in the vicinity of the CRISPR locus, represses the expression of bacterial lipoprotein genes, which is required for bacterial virulence [78,79]. The scaRNA contains a diverged repeat that enables complex formation with tracrRNA by base pairing with the antirepeat, and a 15-bp sequence complementary to the 5′ untranslated region of the lipoprotein operon, which is employed to recognize the target and deliver Cas9. Importantly, it has been shown that the extent of complementarity between the guide RNA and the target regulates Cas9 function [23]. The short duplex formed by scaRNA engenders transcription repression, whereas the longer duplex formed by crRNA triggers target cleavage (Fig 3) [23]. Comparative genomic analysis suggests that scaRNA and, by inference, exaptation of CRISPR for regulation of gene expression is common in type II CRISPR systems [80].

A distinct, even more surprising form of exaptation of a repeat has been discovered in the archaeon *Haloarcula hispanica* and predicted to operate also in related species [22]. The subtype I-B CRISPR locus of these archaea was found to encode a unique TA module that consists of 2 small RNA, the first all-RNA TA module to be discovered. The toxin RNA (CRISPR-resembling toxin, creT) arrests cell growth by binding to the ribosome where it impedes translation by sequestering a rare tRNA. The antitoxin RNA (CRISPR-resembling antitoxin, creA) consists of a repeat-like sequence and a spacer-like sequence that is partially complementary to the *creT* promoter, to which it binds as a complex with Cascade, and represses the toxin expression (Fig 3). This elaborate mechanism safeguards *cas* genes from disruption and deletion, making the host addicted to the CRISPR locus, because abrogation of the genes encoding the Cascade subunits (without disrupting *creTA*, which is a small target) unleashes the toxin.

A different type of repeat exaptation was discovered in the V-K and I-F CASTs (see the previous section). For RNA-guided transposition, both these systems employ a “delocalized crRNA” that is encoded near the transposon end, separately from the CAST CRISPR array [70,81]. The delocalized crRNA consists of a partial copy of the repeat and a spacer-like sequence matching a tRNA to home the transposon near the corresponding tRNA gene. Notably, these CASTs do not utilize the array for homing. Given that the spacers in the CAST array often target other MGE, the CASTs seem to perform 2 distinct functions, namely RNA-guided transposition via the delocalized crRNA and inter-MGE competition via the CRISPR array.

The cases of repeat exaptation discussed above are biologically diverse but all seem to follow the same evolutionary scenario. This series of events involves ectopic duplication of a repeat followed by partial sequence divergence. The crRNA-like transcript containing such a derived repeat forms a complex with a CRISPR effector, targeting it to a sequence recognized by the spacer-like portion of the CRISPR-like RNA. Importantly, these derived repeats located outside CRISPR arrays are not easy to detect in genome analysis as illustrated by the delocalized crRNA and by scaRNA, which were not detected in the initial analyses of the CASTs and type II loci, respectively. Along similar lines, regulatory functions of noncanonical CRISPR RNAs typically rely on partial complementary of the spacer-like sequence and the target, which can serve a toggle between target cleavage and expression regulation [23]. We do not know how many small RNAs containing derived variants of the repeats are lurking around CRISPR loci, and to identify these, dedicated, sophisticated bioinformatic approaches are required as demonstrated by the search for scaRNAs [80].

The final case we discuss further exemplifies the “guns for hire” phenomenon. A search for CRISPR components encoded in MGE led to the unexpected identification of CRISPR miniarrays (typically, 2 repeats flanking a spacer; Fig 3) in many bacterial and archaeal viruses [17]. The repeats in these miniarrays are identical to the repeats in CRISPR arrays of the host, suggesting that miniarrays hijack the host CRISPR machinery. Strikingly, most of the spacers in these miniarrays target other viruses, primarily, those closely related to the miniarray-encoding virus, leading to the hypothesis that miniarrays are involved in intervirus competition [17]. This hypothesis indeed has been validated in experiments with 2 competing archaeal viruses [82]. In addition to the miniarrays, some viruses encode single repeat units that were recognized by identity to the host repeats [17]. The functions of such solo repeat copies remain obscure, but inhibition of the host CRISPR activity seems an attractive possibility.

## Conclusions

The extensive study of CRISPR systems over the last decade or so has revealed remarkable diversity and functional plasticity that are manifested both in the variety of immunity mechanisms and in the exaptation of CRISPR systems and their components for functions different from adaptive immunity or even from any form of defense. At a higher level of generalization, evolution of CRISPR systems seems to have involved 2 phases that occurred convergently in the 2 classes and in some of the types and subtypes (Fig 3). The first phase is the initial complexification via accretion of additional proteins and domains around the core that is typically derived from MGE. The second phase involves simplification and partial degradation, specifically the recurrent loss or inactivation of the components required for interference. In molecular terms, most of the derived CRISPR forms function along the same universal principle, namely the utilization of guide RNAs to direct protein complexes to their sites of action. However, some of the most highly derived variants might have departed from RNA-guided mechanisms. From the functional point of view, a key trend is “guns for hire,” shuttling of RNA-guided systems between MGE and their prokaryotic hosts that often involves reductive evolution. The derived CRISPR variants are typically limited in their spread to relatively narrow groups of prokaryotes or MGE, suggesting that they evolved comparatively recently and are likely to be engaged in specialized functions. Furthermore, often, these systems are hosted by “exotic” bacteria or archaea, making their biological characterization a challenge. Beyond reasonable doubt, many such CRISPR derivatives remain to be discovered. Although the major advances of CRISPR research over the last decade have led to the elucidation of the core mechanisms, the true functional complexity of CRISPR systems and especially the aspects of microbial biology that drive its evolution remain largely unexplored. The study of CRISPR diversity will be a source of fascinating discoveries for years to come.

## Abbreviations

ABI: abortive infection
CARF: CRISPR-associated Rossmann fold
CAST: CRISPR-associated transposase
cOA: cyclic oligoadenylate
crRNA: CRISPR RNA
creA: CRISPR-resembling antitoxin
creT: CRISPR-resembling toxin
HRAMP: haloarchaeal RAMP
ICE: integrative conjugative element
L-tracrRNA: long-form tracrRNA
MGE: mobile genetic element
OMEGA: Obligate Mobile Element Guided Activity
PCD: programmed cell death
RAMP: repeat-associated mysterious protein
RRM: RNA recognition motif
scaRNA: small CRISPR-associated RNA
ssDNA: single-stranded DNA
ssRNA: single-stranded RNA
S-tracrRNA: short-form tracrRNA
TA: toxin–antitoxin
tracrRNA: *trans*-acting crRNA
